# Anti-quorum Sensing and Anti-biofilm Activity of *Delftia tsuruhatensis* Extract by Attenuating the Quorum Sensing-Controlled Virulence Factor Production in *Pseudomonas aeruginosa*

**DOI:** 10.3389/fcimb.2017.00337

**Published:** 2017-07-26

**Authors:** Vijay K. Singh, Avinash Mishra, Bhavanath Jha

**Affiliations:** Marine Biotechnology and Ecology Division, CSIR-Central Salt and Marine Chemicals Research Institute Bhavnagar, India

**Keywords:** anti-biofilm, anti-quorum, microarray, quorum network, quorum quenching, quorum sensing, virulence factors

## Abstract

Multidrug-resistance bacteria commonly use cell-to-cell communication that leads to biofilm formation as one of the mechanisms for developing resistance. Quorum sensing inhibition (QSI) is an effective approach for the prevention of biofilm formation. A Gram-negative bacterium, *Delftia tsuruhatensis* SJ01, was isolated from the rhizosphere of a species of sedge (*Cyperus laevigatus*) grown along the coastal-saline area. The isolate SJ01 culture and bacterial crude extract showed QSI activity in the biosensor plate containing the reference strain *Chromobacterium violaceum* CV026. A decrease in the violacein production of approximately 98% was detected with the reference strain *C. violaceum* CV026. The bacterial extract (strain SJ01) exhibited anti-quorum sensing activity and inhibited the biofilm formation of clinical isolates wild-type *Pseudomonas aeruginosa* PAO1 and *P. aeruginosa* PAH. A non-toxic effect of the bacterial extract (SJ01) was detected on the cell growth of the reference strains as *P. aeruginosa* viable cells were present within the biofilm. It is hypothesized that the extract (SJ01) may change the topography of the biofilm and thus prevent bacterial adherence on the biofilm surface. The extract also inhibits the motility, virulence factors (pyocyanin and rhamnolipid) and activity (elastase and protease) in *P. aeruginosa* treated with SJ01 extract. The potential active compound present was identified as 1,2-benzenedicarboxylic acid, diisooctyl ester. Microarray and transcript expression analysis unveiled differential expression of quorum sensing regulatory genes. The key regulatory genes, *LasI, LasR, RhlI*, and *RhlR* were down-regulated in the *P. aeruginosa* analyzed by quantitative RT-PCR. A hypothetical model was generated of the transcriptional regulatory mechanism inferred in *P. aeruginosa* for quorum sensing, which will provide useful insight to develop preventive strategies against the biofilm formation. The potential active compound identified, 1,2-benzenedicarboxylic acid, diisooctyl ester, has the potential to be used as an anti-pathogenic drug for the treatment of biofilm-forming pathogenic bacteria. For that, a detailed study is needed to investigate the possible applications.

## Introduction

The biggest challenge for the healthcare sector is drug resistance in pathogenic bacteria. The efficiency of antibiotics against pathogenic bacteria is currently decreasing because of the emergence of multidrug-resistance (Adonizio et al., 2008). Biofilm formation is one of the mechanisms, used by bacteria for developing such resistance (Fuqua and Greenberg, 1998). It is well-established that curing of diseases caused by biofilm-forming bacteria requires prolonged treatment, which may also lead to antibiotic resistance due to high evolutionary pressure. The biofilm formation is controlled by cell-to-cell communication, which is widely known as quorum sensing. The inhibition of quorum sensing is one of the methods among the different strategies deployed to control biofilm forming microorganisms without causing drug resistance (Singh et al., 2013, 2016b). In recent years, several anti-quorum sensing compounds were reported in plants and microbes (Choo et al., 2006; Adonizio et al., 2008; Ni et al., 2009; Kalia and Purohit, 2011; Kalia, 2012).

The ubiquitous gram-negative bacterium *Pseudomonas aeruginosa* is an opportunistic pathogen, having a wide range of hosts such as insects, plants, animals, and humans (Rahme et al., 2000; Vandeputte et al., 2010). The bacterium *P. aeruginosa* causes very severe infection in immunocompromised patients (Driscoll et al., 2007; Vandeputte et al., 2010; Sarabhai et al., 2013) and is responsible for about 57% of all nosocomial infections (Oncul et al., 2009; Sarabhai et al., 2013).

It was observed that *P. aeruginosa* uses a range of virulence factors and multiple mechanisms, including biofilm formation, to successfully infect a diverse range of hosts and to protect itself from environmental stress and antibiotics (Driscoll et al., 2007; Vandeputte et al., 2010; Lee and Zhang, 2015). Quorum sensing controls the virulence factors and biofilm formation of *P. aeruginosa*. Therefore, anti-quorum sensing strategies could be a potential target to prevent *P. aeruginosa* infection.

The rhizosphere, a region of soil that surrounds the plant roots, possess a diverse bacterial community that containing molecules with both quorum sensing and quorum quenching activities (Christiaen et al., 2011), including anti-biofilm activity against *P. aeruginosa* (Christiaen et al., 2014). The anti-quorum sensing activity of *Acinetobacter* sp. strain C1010 (isolated from cucumber rhizosphere) was evaluated and found to degrade the acyl-homoserine lactones (AHLs) produced by *P. chlororaphis* O6 (Kang et al., 2004). A large number of AHL-degrading bacteria, including *Sphingomonas* sp. and *Bosea* sp., were isolated from the tobacco rhizosphere (D'Angelo-Picard et al., 2005). The bacteria *Acinetobacter* (GG2), *Burkholderia* (GG4), and *Klebsiella* (Se14) isolated from the ginger rhizosphere also showed AHL-degrading activity (Chan et al., 2011). Bacterial consortia isolated from the rhizosphere of potato contained anti-quorum sensing and plant growth promoting potential (Cirou et al., 2007). To date, however, there is no report on rhizospheric bacteria with anti-quorum sensing and anti-biofilm activity from the saline ecosystem.

In the present study, the rhizosphere of a monocot *Cyperus laevigatus*, a species of sedge from the coastal saline area, was explored and the bacterium *Delftia tsuruhatensis* SJ01 was isolated. Members of the *Delftia* genus are Gram-negative, aerobic, rod-shaped and motile bacteria comprised of five species: *Delftia acidovorans* (Wen et al., 1999), *D. tsuruhatensis* (Shigematsu et al., 2003), *Delftia lacustris* (Jørgensen et al., 2009), *Delftia litopenaei* (Chen et al., 2012), and *Delftia deserti* (Li et al., 2015). The coral-associated bacterial strain *D. tsuruhatensis* from the Gulf of Mannar was reported for its anti-quorum sensing activity. However, a detailed study and the identification of compounds has still not been performed (Bakkiyaraj et al., 2012, 2013). The isolated bacterium was explored for anti-quorum sensing and anti-biofilm potential. The active fraction was identified, regulatory key genes were studied, and a possible mechanism was inferred.

## Materials and methods

### Isolation and screening of bacteria

A monocot, *C. laevigatus*, growing luxuriantly in the wet coastal areas of New-port, Bhavnagar, India (Latitude N 21° 45.124″, Longitude E 72° 13.579″), was collected. Bacteria were isolated from rhizosphere using a standard method, and axenic cultures were made for each isolate. Isolated axenic cultures were subjected to the screening of anti-quorum sensing activity using the reference strain *Chromobacterium violaceum* (CV026), cinnamaldehyde (Sigma-Aldrich, USA) as a positive control and methanol as a negative control in a plate-based bioassay (Singh et al., 2013). Bacterial isolates showing quorum sensing inhibition (QSI) activity were selected and checked further for antibacterial activity on Mueller-Hinton agar (MHA), along with tobramycin, which used as a positive control (Choo et al., 2006). A bacterial isolate showing promising positive QSI and negative anti-bacterial activities was selected further. The QSI and anti-bacterial activities of the selected isolate were repeated five times independently.

### Identification of bacteria and fatty acid methyl ester profiling

Genomic DNA of selected bacteria was isolated, and the 16S rRNA gene amplified with universal primers fD1-5′-AGA GTT TGA TCC TGG CTC AG-3′ and rP2-5′-ACG GCT ACC TTG TTA CGA CTT-3′ (Weisburg et al., 1991) and optimized PCR conditions (Keshri et al., 2013, 2015). The PCR product was purified, sequenced (M/s Macrogen Inc., South Korea) and subjected to BLAST analysis. Phylogenetic analysis was performed using MEGA (Molecular Evolutionary Genetics Analysis) version 6.0 software (Tamura et al., 2013). The phylogenetic tree was reconstructed using neighbor-joining methods (Saitou and Nei, 1987), bootstrap analysis was performed (Felsenstein, 1985), and evolutionary distances were determined using maximum composite likelihood algorithms (Tamura et al., 2004). The bacterial isolate was identified as *D. tsuruhatensis* strain SJ01, and the 16S rRNA gene sequence was deposited in the NCBI GenBank (KX130769).

Fatty acid methyl ester (FAME) profiling of identified bacteria was performed using Microbial Identification System (MIDI; Microbial ID) coupled with gas chromatography (GC system-6850, Agilent Technologies, USA). For whole cell fatty acid methyl ester profiling, the bacteria were grown on tryptic soy yeast agar for 24 h at 30°C, and fatty acid methyl esters were prepared according to the instruction manual of the Microbial Identification System (MIDI; Microbial ID). Peaks were identified and matched with RTSBA6 6.10 database (Jha et al., 2015).

### Preparation of bacterial extract

Bacterial culture (*D. tsuruhatensis* strain SJ01, 500 ml in nutrient broth, NB), grown for 48 h, 180 rpm at 30°C was centrifuged for 15 min. at 10,000 × g, 4°C, and the supernatant was collected in a flask. The supernatant was filtered through 0.45 and 0.22 μm vacuum filters for the complete removal of bacterial cells. The filtrate was extracted twice with an equal volume of ethyl acetate. Ethyl acetate extract was evaporated to dryness under vacuum in a rotary evaporator (Büchi, Switzerland) and dissolved in methanol for further studies (Nithya et al., 2010).

### Anti-quorum sensing activity

The anti-quorum sensing activity of a methanolic extract of bacteria was tested by quantifying violacein (Choo et al., 2006). In brief, 1 ml of the freshly grown (OD_600nm_ 0.7) reference strain *C. violaceum* (CV026) was added to 20 ml NB Hi-veg media (Hi-media, India) containing hexonyl homoserine lactone (0.0625 μg/ml) and different concentrations of bacterial extract (0.01, 0.02, 0.03, 0.04, 0.05, 0.075, or 0.1 mg/ml). Cultures without extract and with methanol were considered the control and negative control, respectively. All cultures (controls and experimental) were incubated for 24 h at 30°C and 180 rpm (Choo et al., 2006). One milliliter of overnight grown culture from each flask was centrifuged 16,000 × g for 10 min, and the pellet containing violacein (produced by CV026) was suspended in 1 ml of dimethylsulfoxide (DMSO). The solution was centrifuged at 16,000 × g for 10 min to remove cell debris and absorbance was read at 585 nm in a microplate reader (Spectra Max Plus, USA).

### Biofilm formation assay

A measure of 200 μl of overnight grown cultures (OD_600nm_ 0.1) of clinical isolates *P. aeruginosa* PAO1 (ATCC 15692) or *P. aeruginosa* PAH (by courtesy from Govt. Medical College, Bhavnagar; Goswami et al., 2011) was added to a 96-well microtiter plate with different concentrations of bacterial (strain SJ01) extracts (0.01, 0.02, 0.03, 0.04, 0.05, 0.075, and 0.1 mg/ml). The plate was incubated at 37°C, 100 rpm for 24 h, after which the growth of bacteria was measured at 600 nm and colony forming units (CFU) were also determined. Wells were washed after removing planktonic bacterial cells, dried and stained with 1% crystal violet. Excess dye was taken out after 20 min, wells were washed (with sterile distilled water), 200 μl ethanol (aqueous 96%) was added, and absorbance was measured at 590 nm (Andersson et al., 2009; Singh et al., 2013; Kavita et al., 2014). The experiments were performed thrice with five replicates each.

### Fluorescence microscopy

Cell viability within the biofilm was examined at different time points (24, 48, and 72 h) and compared with the control (Singh et al., 2013). Cells inhabiting the biofilm were stained with a fluorescent dye using the FilmTracer™Live/Dead® Biofilm Viability Kit (Invitrogen, USA) following manufacturer's instructions and visualized under an epi-fluorescence microscope (Axio Imager, Carl Zeiss AG, Germany).

### Scanning electron microscopy

The effect of bacterial extract (SJ01) on biofilm formation was visualized by scanning electron microscopy (SEM; Andersson et al., 2009; Singh et al., 2013). Biofilms of *P. aeruginosa* PAO1 and *P. aeruginosa* PAH, grown on glass coverslips (11 mm) submerged in nutrient broth with (0.1 mg/ml) or without bacterial extract were gently washed with 0.9% NaCl to remove planktonic cells. Samples were kept in 2.5% glutaraldehyde for 20 min followed by 4% OsO_4_ in 0.1 M phosphate buffer for 30 min. Samples were dehydrated with a gradient ethanol series (10–95%) for 10 min. The dried biofilms were coated with gold and visualized under a scanning electron microscope (SEM, LEO series VP1430, Germany).

### Atomic force microscopy

For atomic force microscopy (AFM), biofilms developed on glass coverslips were rinsed gently with phosphate buffer saline (pH 7.4) and kept in a desiccator for drying completely. The biofilm was scanned under AFM (NT-MDT, Russia) in a semi-contact mode at the speed of 1 Hz (Oh et al., 2009; Nithya et al., 2010). The surface bearing index (Sbi), core fluid retention index (Sci), valley fluid retention index (Svi), kernel roughness depth (Sk), reduced peak height (Spk), reduced valley depth (Svk), average roughness (Sa), root mean square (Sq), surface skewness (Ssk), coefficient of kurtosis (Ska), and surface area ratio (Sdr) were calculated.

### Bacterial motility assay

Bacterial extract (SJ01) was tested on the swarming and swimming motility of *P. aeruginosa*. For the swarming motility assay, *P. aeruginosa* strains were spotted on a plate containing BM2 swarming medium (62 mM PBS at pH 7, 2 mM MgSO_4_, 10 μM FeSO_4_, 0.4% glucose, 0.1% casamino acids, and 0.5% agar) supplemented with (0.1 mg/ml) or without extract (Overhage et al., 2007). For the swimming motility assay, *P. aeruginosa* strains were spotted on a plate containing tryptone broth (10 g/l tryptone, 5 g/l NaCl, and 0.3% agar) supplemented with (0.1 mg/ml) or without extract (Rashid and Kornberg, 2000). Plates were analyzed after incubation of 24 h at 37°C.

### Virulence factor analysis

The effect of bacterial extracts (SJ01; 0.1 mg/ml) was studied on the production of virulence factors of reference *P. aeruginosa* strains by quantifying pyocyanin and rhamnolipid, and analyzing elastase and protease activities. Briefly, *P. aeruginosa* PAO1 and *P. aeruginosa* PAH were grown overnight in 5 ml of PB medium (20 g/l peptone, 1.4 g/l MgCl_2_ and 10 g/l K_2_SO_4_) supplemented with extract of strain SJ01 (0.1 mg/ml) and without extract (control) at 37°C (180 rpm). The culture was centrifuged at 10,000 × g for 10 min, and pyocyanin was extracted first from the supernatant in 3 ml of chloroform, followed by 1 ml of 0.2 N HCl. The absorbance was measured spectrophotometrically at 520 nm (Essar et al., 1990).

For rhamnolipid, reference strains (*P. aeruginosa*) were grown in nutrient broth supplemented with bacterial extract (SJ01; 0.1 mg/ml) or without extract (control). The culture was centrifuged at 10,000 × g for 10 min, supernatants were collected, acidified with HCl (to pH 2) and absorbance was measured at 570 nm (McClure and Schiller, 1992). Supernatants (750 μl) of overnight grown (with 0.1 mg/ml or without extract of strain SJ01) *P. aeruginosa* were incubated with 250 μl elastin Congo-red solution (5 mg/ml in 0.1 M tris-HCl pH 8; 1 mM CaCl_2_) at 37°C, 180 rpm for 16 h. After incubation, the mixture was centrifuged at 3,000 × g for 10 min, and absorbance was measured at 490 nm for elastase activity (Zhu et al., 2002). For protease activity, supernatant (400 μl) was incubated with an equal volume of 2% azocasein solution (prepared in 50 mM phosphate buffer saline, pH 7) at 37°C for 1 h. The reaction was stopped by adding 500 μl of 10% trichloroacetic acid (TCA), and reaction mix was centrifuged at 8,000 g for 5 min to remove residual azocasein. The absorbance of the supernatant was read at 400 nm (Adonizio et al., 2008).

### Fractionation and identification of active compound

Bacterial extract (*D. tsuruhatensis* SJ01) was fractionated by the solid phase extraction (SPE) method using different cartridges (non-polar C18, polar SI, anion exchanger DAE and cation mixed Plexa PCX) and each fraction was screened for anti-quorum sensing activity. The positive fraction was further analyzed, and an active compound was identified by GC-MS. Briefly, crude bacterial extract (1 ml) was loaded to the preconditioned (by 5 ml methanol, 10 ml water and 5 ml acidified water pH 2.0) SPE cartridges (Agilent, USA). The elution was performed with a different concentration of 1 ml methanol (20, 40, 60, 80, and 100% v/v in water) and different fractions were collected (Singh et al., 2013). Each fraction was screened for plate based anti-quorum sensing activity (as described above) using the reference strain *C. violaceum* (CV026). The positive fraction was subjected to GC-MS (GC-2010, Shimadzu, Japan) and the identification of compounds was done by comparing the mass spectra with the reference mass spectra library. The mass of the fractionated compound identified was further confirmed by electrospray ionization mass spectrometry (ESI-MS; Q-Tof micro TM, Micromass, UK), performed in a positive mode.

### Microarray and expression analysis

Differential expression of regulatory genes of reference strain *P. aeruginosa* PAO1, involved in the quorum sensing was analyzed using microarray. Total RNA was isolated from reference strain *P. aeruginosa* PAO1, grown with or without bacterial extracts (0.1 mg/ml) using TRI reagent (Sigma, USA). Total RNA was quantified, and 10 μg RNA was converted to cDNA, befor being fragmented and labeled by following the GeneChip® *P. aeruginosa* PAO1 genome array user manual (Affymetrix, USA). Labeled cDNAs were hybridized with the *P. aeruginosa* genome array gene chip (containing total 5,886 gene probes), and then washed and stained (Singh et al., 2016a). Hybridized chips were scanned (Scanner 3000 7G, Affymetrix, USA), processed and analyzed using the expression console and the transcriptome analysis console (Affymetrix, USA). Microarray analysis was performed in duplicate (*n* = 2) and genes exhibiting significant fold expression (ANOVA *p* < 0.05) were considered for the study. All microarray data are available with Array-Express accession number E-MTAB-5693. For expression profiling, key regulatory genes (*LasI, LasR, RhlI*, and *RhlR*) were selected. Total RNA was extracted from control and treated *P. aeruginosa* (PAO1 and PAH strains) converted to cDNA and then quantitative real-time PCR was performed (Wang et al., 2005). A melt curve analysis was also done for the validation of specificity of the qRT-PCR reaction, and the relative fold expression change was calculated using the CT method (Livak and Schmittgen, 2001). The 16S rRNA gene was used as a reference gene (Wang et al., 2005).

## Results

### Isolation and screening of bacteria for anti-quorum sensing activity

**Figure 1:**
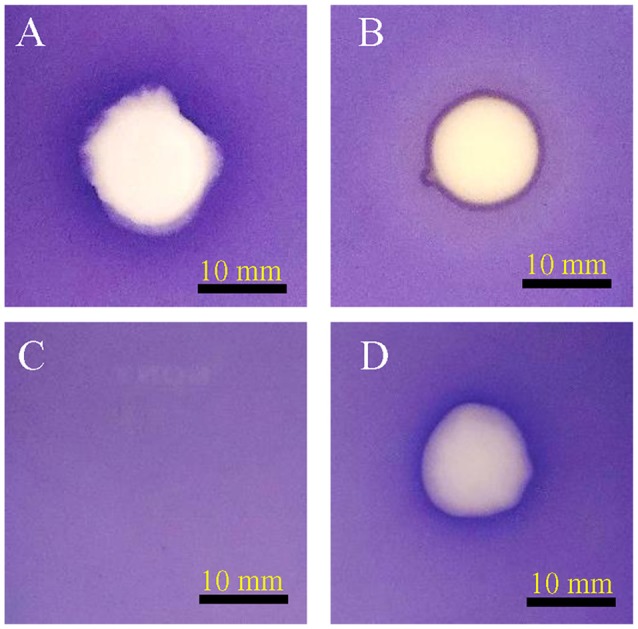
Anti-quorum sensing activity of isolate SJ01. The biosensor plates containing reference strain *C. violaceum* CV026 were spotted with **(A)** cinnamaldehyde, **(B)** SJ01 axenic culture, **(C)** methanol, and **(D)** crude bacterial (SJ01) extract. Cinnamaldehyde was used as a positive control. The isolate SJ01 and its extract showed the anti-quorum sensing activity and a clear white opaque zone of inhibition.

A total of 56 bacterial axenic cultures were obtained from the rhizosphere of *C. laevigatus* L., of which two axenic cultures showed anti-quorum sensing activity in a plate-based bioassay. The isolate SJ01 showed promising anti-quorum sensing activity and a clear white opaque zone of inhibition was observed in the biosensor plate containing reference strain *C. violaceum* CV026 (Figure 1). Furthermore, the bacterial crude extract also showed QSI, whereas the zone of inhibition was not detected with the negative control (methanol). The disc diffusion antibacterial assay confirmed that selected bacterial isolates did not show antibacterial activity against the reference strain *C. violaceum* CV026 (Figure S1).

### Identification of bacteria, fatty acid methyl ester profiling, and phylogenetic analysis

The 16S rRNA gene sequence (accession no. KX130769) of the selected bacterial isolate showed 99% similarity to *D. tsuruhatensis*, with 100% query coverage; therefore, this was designated *D. tsuruhatensis* SJ01. The phylogenetic tree reconstructed using the neighbor-joining algorithm shows the taxonomic position of identified bacterium with other species (Figure S2). The whole cell fatty acid profiling of the bacterium *D. tsuruhatensis* SJ01 revealed the abundance of C_16:0_ fatty acids (Figure S3).

### *Delftia tsuruhatensis* SJ01 extract shows anti-quorum sensing activity by inhibiting violacein production

**Figure 2:**
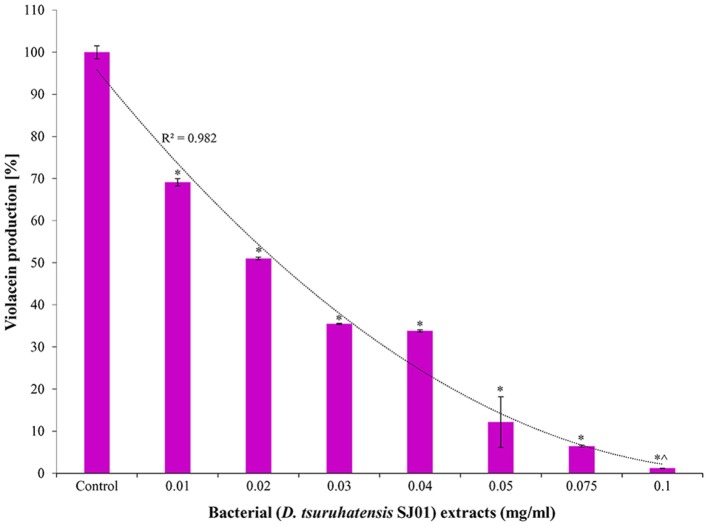
Effect of different concentration of *D. tsuruhatensis* SJ01 extract on violacein production. Different concentration of bacterial extract (0.01−0.1 mg/ml) was used to quantify the inhibition of violacein, an indicator of quorum sensing activity. Cultures without extract were considered as a control. ^*^Indicates significant differences from the control at *P* < *0.05* and ^∧^ indicates maximum significant differences from the control at *P* < *0.05*.

The bacterium *D. tsuruhatensis* SJ01 and its methanolic extract showed anti-quorum sensing activity with the reference strain on a biosensor plate. Different concentrations of bacterial extract were used to quantify the inhibition of violacein, an indicator of quorum sensing activity (Figure 2). The violacein production decreased concomitantly with the increasing concentration of the extract, and about 98% inhibition was observed with 0.1 mg/ml extract.

### *Delftia tsuruhatensis* SJ01 extract inhibits biofilm formation

**Figure 3:**
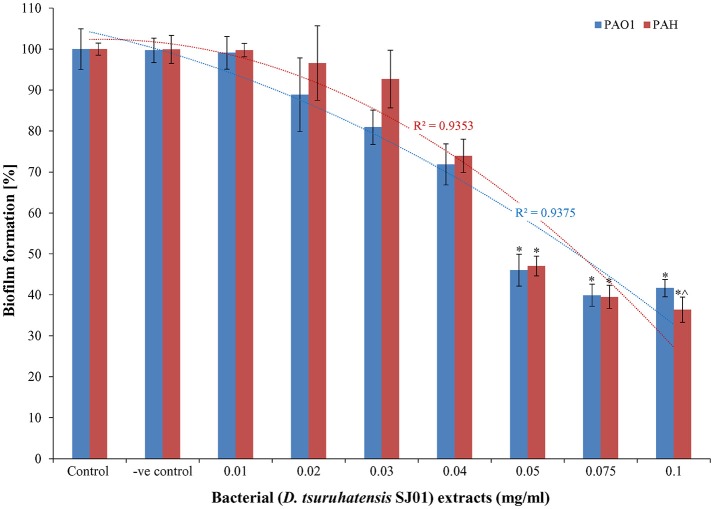
The antibiofilm activity of *D. tsuruhatensis* SJ01 extract. Different concentration of bacterial extracts (0.01−0.1 mg/ml) was tested against wild-type, widely used biofilm forming reference strain *P. aeruginosa* strains. Tests without extract and with methanol were considered as control and negative control, respectively. ^*^Indicates significant differences from the control at *P* < *0.05* and ^∧^ indicates maximum significant differences from the control at *P* < *0.05*.

The anti-biofilm activity of the extract (*D. tsuruhatensis* SJ01) was tested against the wild-type, widely used biofilm forming clinical isolate *P. aeruginosa* PAO1 and a local clinical isolate *P. aeruginosa* PAH. The biofilm formation decreased concurrently in both reference strains with increasing concentration of bacterial extracts (Figure 3). About 60–64% inhibition of the biofilm formation was observed with 0.1 mg/ml extract. The possibility of an inhibitory effect of *D. tsuruhatensis* SJ01 extract on the growth of reference strains (*P. aeruginosa*) was also analyzed (Figures S4, S5). No significant effect was observed on the planktonic growth of *P. aeruginosa* in the presence of different concentration of bacterial extracts (0.01–0.1 mg/ml). Further, the disc diffusion antibacterial assay performed with SJ01 extract confirmed that bacterial extract did not show antibacterial activity against the clinical isolates *P. aeruginosa* (Figure S6).

### Fluorescence microscopy analysis confirms that biofilm inhabiting viable cells

**Figure 4:**
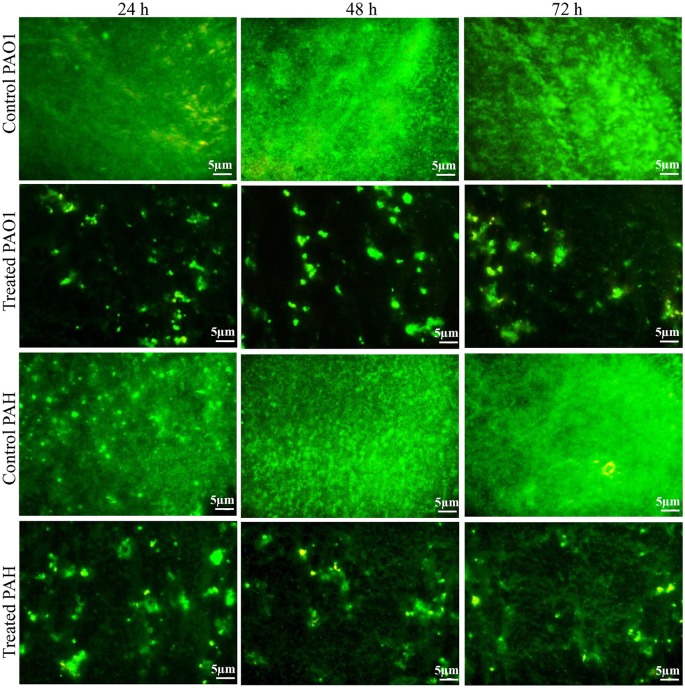
Epi-fluorescence micrographs of biofilms developed by *P. aeruginosa*. The effect of the bacterial extract (0.1 mg/ml) on the viability of reference *P. aeruginosa* strains in the biofilm was examined at different time points (24, 48, and 72 h) and compared with control. The dead bacterial cells were labeled with propidium iodide whereas live cells stained with SYTO 9, which produced red and green fluorescence, respectively.

The effect of the bacterial extract on the viability of the reference strain in the biofilm (24–72 h) was studied with an epi-fluorescence microscope (Figure 4). The dead *P. aeruginosa* cells were labeled with propidium iodide whereas live cells stained with SYTO 9, which produced red and green fluorescence, respectively. Less attachment of *P. aeruginosa* cells to the surface was observed even up to 72 h in the treated biofilm compared to control, and an insignificant number of dead cells was detected in the biofilms.

### *Delftia tsuruhatensis* SJ01 extract disrupts the architecture of the biofilm

**Figure 5:**
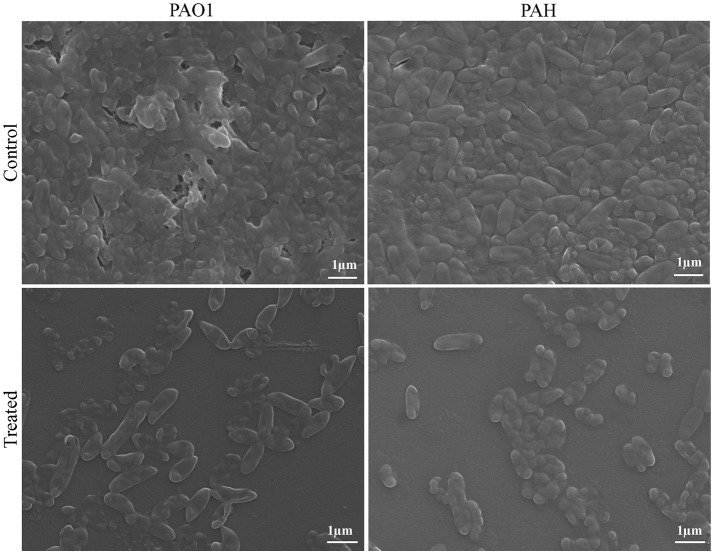
SEM images of biofilms developed by *P. aeruginosa*. SEM images illustrating the effect of bacterial extract (0.1 mg/ml) on biofilm formation. A well-grown biofilm along with adhering bacterial cells was observed in control (normal biofilm developed by *P. aeruginosa*), whereas dispersed bacterial cells were observed in treated samples.

**Figure 6:**
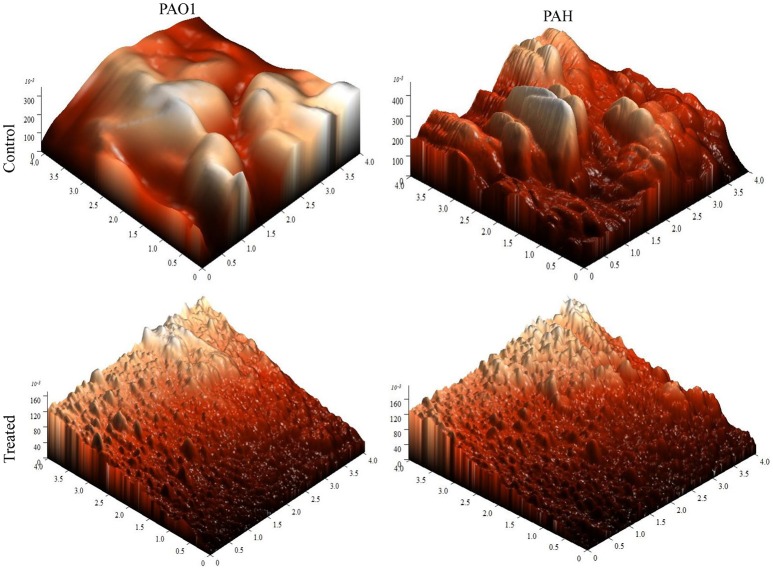
AFM images illustrating the effect of *D. tsuruhatensis* SJ01 extract on *P. aeruginosa* biofilms. AFM showed a disrupt surface topology and height distribution profile of the biofilm developed by reference *P. aeruginosa* strains in the presence of bacterial extract (0.1 mg/ml) compared to the control biofilm.

The topology of the biofilm developed by *P. aeruginosa* and the effect of *D. tsuruhatensis* SJ01 extract on it was analyzed by SEM and AFM. A well-grown biofilm along with adhering bacterial cells was observed in controls (normal biofilm developed by *P. aeruginosa*) in the SEM analysis, whereas dispersed bacterial cells were observed in treated samples (Figure 5). Similarly, AFM clearly showed the disrupted surface topology and height distribution profile of the biofilm developed in the presence of *D. tsuruhatensis* SJ01 extract compared to the control biofilm (Figure 6). The surface bearing indices, roughness analysis, and functional parameters based on the linear material ratio curve showed alterations of the biofilm developed in treated samples (Table 1).

**Table 1:** Statistical analysis of biofilm analyzed by atomic force microscopy (AFM).

**Statistical parameters**	**Control PAO1**	**Treated PAO1**	**Control PAH**	**Treated PAH**
Root Mean Square (Sq)	0.06	0.04	0.08	0.04
Surface Bearing Index (Sbi)	2.22	1.06	0.74	0.94
Core Fluid Retention Index (Sci)	1.44	1.49	1.76	1.68
Valley Fluid Retention Index (Svi)	0.09	0.07	0.08	0.07
Kernel roughness depth (Sk)	0.20	0.12	0.20	0.12
Reduced peak height (Spk)	0.02	0.02	0.10	0.03
Reduced valley depth (Svk)	0.06	0.01	0.05	0.01
Roughness Average (Sa)	0.05	0.03	0.06	0.03
Surface skewness (Ssk)	0.22	0.09	0.41	0.26
Coefficient of kurtosis (Ska)	2.47	2.02	3.07	2.16
Surface Area Ratio (Sdr), %	0.03	0.01	0.09	0.03

### *Delftia tsuruhatensis* SJ01 extract shows inhibitory effect on the motility of *P. aeruginosa*

**Figure 7:**
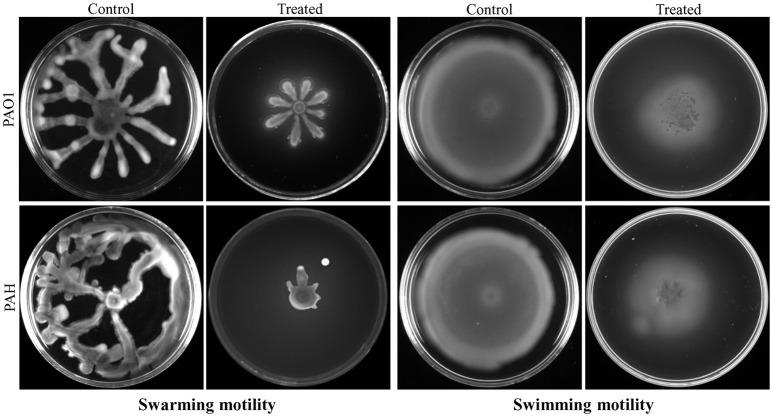
Study of cell motility of *P. aeruginosa*. The effect of bacterial extract (*D. tsuruhatensis* SJ01) on the swarming and swimming motility of reference *P. aeruginosa* strains was studied. *P. aeruginosa* was spotted on a plate supplemented with (0.1 mg/ml) or without extract. Plates were analyzed after incubation of 24 h at 37 °C.

Bacterial invasion is a prerequisite for biofilm formation. Therefore, the effect of bacterial extract (*D. tsuruhatensis* SJ01) was studied on the motility of biofilm forming *P. aeruginosa* bacterial cells. It was observed that bacterial extract (0.1 mg/ml) inhibits the swarming and swimming motility of *P. aeruginosa* strains in the plate assay (Figure 7). The extract reduced flagellum driven motility of *P. aeruginosa* in the treated sample compared to the control.

### *Delftia tsuruhatensis* SJ01 extract relegates the virulence activities

**Figure 8:**
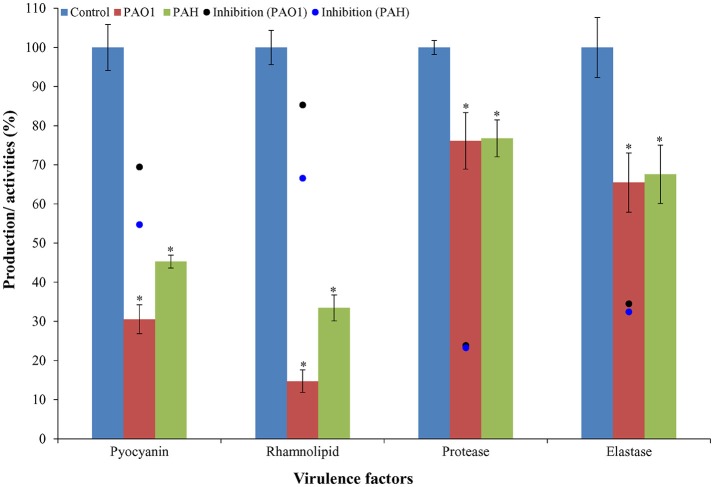
Effect of *D. tsuruhatensis* SJ01 extract on the virulence factors of *P. aeruginosa*. The effect of bacterial extracts (SJ01; 0.1 mg/ml) was studied on the production of virulence factors of reference *P. aeruginosa* strains by quantifying pyocyanin and rhamnolipid, and analyzing elastase and protease activities. ^*^Indicates significant differences from the control at *P*< *0.05*.

It was observed that bacterial extract (*D. tsuruhatensis* SJ01) reduced the production of virulence factors; pyocyanin and rhamnolipid (Figure 8). Pyocyanin production decreased about 70 and 55% in PAO1 and PAH strains, respectively with the treatment of 0.1 mg/ml bacterial extract. Similarly, rhamnolipid production was also decreased by 85 and 67% in PAO1 and PAH strains, respectively, in the presence of bacterial extract (0.1 mg/ml). The effect *D. tsuruhatensis* SJ01 extract on the elastase and protease activities of cell-free *P. aeruginosa* bacterial culture supernatant were also assessed (Figure 8). About 32–35% decrease in elastase activities was detected for both strains compared to the control. However, about 23–24% inhibition in the protease activity was found in both strains with 0.1 mg/ml bacterial extract compared to untreated samples.

### Identification of quorum sensing inhibitor compound

**Figure 9:**
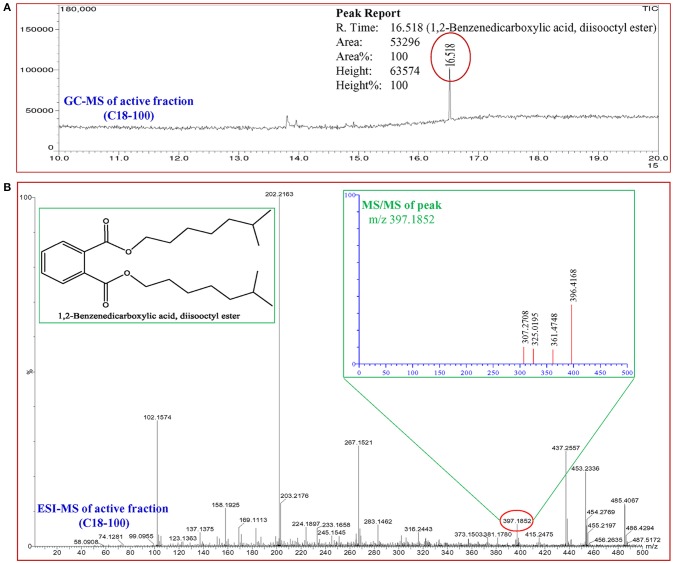
Analysis of active fraction showing quorum sensing inhibition. **(A)** GC chromatograms and **(B)** ESI-MS/MS of the C18-100 active fraction of *D. tsuruhatensis* SJ01 extract and structure of 1,2-benzenedicarboxylic acid, diisooctyl ester (redrawn by ChemBioDraw Ultra 12.0).

In total, five fractions (in 20, 40, 60, 80, and 100% methanol) were collected through each SPE cartridge (non-polar C18, polar SI, anion exchanger DAE, and cation mixed Plexa PCX); all were screened individually for QSI using a biosensor plate containing *C. violaceum* CV026. Fraction (C18-100), collected through the C18 cartridge with 100% methanol, showed a maximum zone of QSI; therefore, this was selected for further characterization. Fraction C18-100 was subjected to GC-MS analysis, and the chromatogram showed a single peak at the retention time 16.518 min (Figure 9). The detected mass spectra showed some resemblance to 1,2-benzenedicarboxylic acid, diisooctyl ester, in the GC-MS library (NIST 27. LB). The calculated (theoretical) or expected molecular mass of compound 1,2-benzenedicarboxylic acid, diisooctyl ester (C_24_H_38_O_4_) is 390.55_._ The molecular mass of the active fraction (C18-100) was further confirmed by ESI-MS. A mass spectral peak, detected at *m/z* 397.1852, was considered the corresponding experimental mass of the active fraction (Figure 9).

### Microarray and transcript expression analyses exhibit differential expression of QS regulatory genes

**Figure 10:**
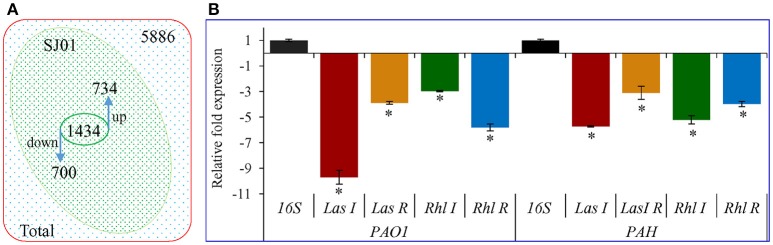
Transcript expression analysis of *P. aeruginosa* treated with *D. tsuruhatensis* SJ01 extract. **(A)** Venn diagram showing genes differentially expressed in *P. aeruginosa* PAO1 as studied by microarray and **(B)** expression profiling of some QSI regulatory genes from *P. aeruginosa* (strains PAO1 and PAH). ^*^Indicates significant differences from the control at *P* < *0.05*.

Differential expression of quorum sensing regulatory genes of reference strain *P. aeruginosa* PAO1 treated with a bacterial fraction (C18-100) containing 1,2-benzenedicarboxylic acid, diisooctyl ester as a probable bioactive compound was analyzed using *P. aeruginosa* PAO1 genome array gene chip. Out of the 5,886 gene probe sets, 1,434 genes were differentially expressed (Table S1; Array-Express accession E-MTAB-5693) and showed at least 2-fold up- (>2) or down-(< −2) expression at *p* < 0.05 (Figure 10). Of these, 734 genes were up-regulated, whereas 700 genes were down-regulated. Some differentially expressed important genes (as observed in microarray analysis) involved in the quorum sensing and general metabolic pathways are listed in Table 2. The microarray scattered plot showed the differential expression of genes; up-regulation of genes was indicated by blue marks whereas green-colored dots represented down-regulation (Figure S7). The quantitative RT-PCR revealed that the genes *LasI, LasR, RhlI*, and *RhlR* were down-regulated in the treated *P. aeruginosa* compared to the control (Figure 10). About, 9.7-, 3.9-, 3-, and 5.9-fold down-regulation of the genes *LasI, LasR, RhlI* and *RhlR*, respectively, was observed in *P. aeruginosa* PAO1 strain. Similarly, 5.7-, 3.1-, 5.2-, and 4-fold decrease in gene expression was found in *P. aeruginosa* PAH strain.

**Table 2:** Selected transcripts that differentially expressed (up- or down- regulated) in *P. aeruginosa* PAO1, treated with bacterial (*D. tsuruhatensis* SJ01) active fraction (C18-100; containing 1,2-benzenedicarboxylic acid, diisooctyl ester) compared with control (untreated PAO1 strain).

**Transcript ID**	**Gene symbol**	**Description**	**Swiss Prot**	**Fold-change**
PA1985	*pqqA*	Pyrroloquinoline quinone biosynthesis protein A	Q9ZAA0	−27.72
PA0998	*pqsC*	Homologous to beta-keto-acyl-acyl-carrier protein synthase	Q9I4X1	−8.94
PA2238	*pslH*	Hypothetical protein	Q9I1N1	−7.13
PA5368	*pstC*	Membrane protein component of ABC phosphate transporter	Q51544	−5.65
PA2236	*pslF*	Hypothetical protein	Q9I1N3	−5.41
PA1988	*pqqD*	Pyrroloquinoline quinone biosynthesis protein D	Q9I2C1	−3.9
PA5070	*tatC*	Transport protein TatC	Q9HUB3	−3.47
PA4225	*pchF*	Pyochelin synthetase	Q9HWG4	−2.79
PA3061	*pelD*	Hypothetical protein	Q9HZE7	−2.46
PA1989	*pqqE*	Pyrroloquinoline quinone biosynthesis protein E	Q9I2C0	−2.31
PA3477	*rhlR*	Transcriptional regulator RhlR	P54292	−2.22
PA2424	*pvdL*	Adaptation/ protection	Q9I157	2.76
PA5373	*betB*	Betaine aldehyde dehydrogenase	Q9HTJ1	2.79
PA1000	*pqsE*	Quinolone signal response protein	–	2.83
PA1003	*mvfR*	Transcriptional regulator	Q9I4X0	3.6
PA3103	*xcpR*	General secretion pathway protein	Q00512	4.14
PA1719	*pscF*	Type III export protein PscF	P95434	4.45
PA2245	*pslO*	Hypothetical protein	–	5.31
PA4205	*mexG*	Hypothetical protein	Q9HWH6	6.49
PA3058	*pelG*	Hypothetical protein	Q9HZF0	7.82
PA4085	*cupB2*	Chaperone protein	Q9HWU3	8.29

*“−” sign means down-regulation*.

## Discussion

Natural products are an imperative source for the discovery of novel therapeutics, and microbes are therefore considered a primary source for drug discovery (Gillespie et al., 2002; Courtois et al., 2003). Biofilm forming bacteria are shown to be resistant toward a broad spectrum of antibiotics and make it difficult to cure biofilm-related infections (Høiby et al., 2010). It has been demonstrated that the social behavior of bacterial life depends on two interrelated phenomena: quorum sensing and biofilm formation (Nadell et al., 2008). Biofilm formation of pathogenic *P. aeruginosa* is controlled by the quorum sensing (QS) regulatory genes, and anti-quorum sensing compounds are explored to inhibit the biofilm formation. These compounds intervene in the QS mechanism and inhibit the expression of virulence factors. Recently, it has been shown that commercially available anti-QS compounds could increase the susceptibility of bacterial biofilm to antibiotics, both *in vitro* and *in vivo* (Brackman et al., 2008). Anti-QS properties have been reported from several rhizospheric bacteria, and *Stenotrophomona rhizosphila* reduced the AHL level (Christiaen et al., 2011). The rhizosphere of different plants (cucumber, tobacco, and ginger) was also exploited to isolate bacteria with anti-quorum sensing activity (Kang et al., 2004; D'Angelo-Picard et al., 2005; Chan et al., 2011). In this study, *D. tsuruhatensis* SJ01 was isolated from the rhizosphere of *C. laevigatus* L. collected from the coastal saline area. Previously, we have demonstrated that *Stenotrophomonas maltophilia*, isolated from *C. laevigatus* rhizosphere, showed quorum quenching and anti-biofilm forming activity (Singh et al., 2013).

Violacein production is a prerequisite for quorum sensing that leads to biofilm formation. A reference strain *C. violaceum* CV026 is well known for the production of violacein in the presence of external AHL and is widely used for quorum sensing studies. Extracts of *D. tsuruhatensis* SJ01 showed anti-QS activity against *C. violaceum* CV026 on biosensor plates (Figure 1) and inhibited violacein production in a concentration-dependent manner (Figure 2). About 98% inhibition of violacein production was detected with 0.1 mg/ml *D. tsuruhatensis* extract. However, it is difficult to compare the results with previous reports because of variation in the extraction methods and other parameters. About 90–94% reduction in the violacein production was reported with 3–4 mg/ml extract of *S. maltophilia* and *Melicope lunu-ankenda* extracts (Tan et al., 2012; Singh et al., 2013). Furthermore, the zone of inhibition was not observed when *D. tsuruhatensis* was spotted onto a plate containing *C. violaceum* culture (Figure S1). This rules out the possibility of antibacterial (*C. violaceum*) activity of *D. tsuruhatensis*. Inhibition of the AHL-dependent quorum sensing mechanism of CV026 (Figure 2) revealed the anti-quorum sensing potential of the extract at very low concentration (0.1 mg/ml).

The extract of *D. tsuruhatensis* SJ01 inhibits the biofilm formation of clinical isolates *P. aeruginosa* PAO1 as well as *P. aeruginosa* PAH (Figure 3) without affecting planktonic growth (Figures S4, S5). Strain PAO1 showed about 15% increase in planktonic cell growth (with a higher concentration of extracts), possibly because of the inability of strains to attach to the surface and subsequently to form a biofilm. This may lead to an increase of planktonic cell growth. However, a detailed study is required to ascertain the exact reason behind it. The viable *P. aeruginosa* cells were observed under epi-fluorescence microscopy (Figure 4) which confirmed that extract (SJ01) does not have a toxic effect on cells within the biofilm (Figure 4). The functional indices of biofilm exhibited physical characteristics (Ţǎlu, 2013). The AFM-based statistical analysis indicated a decrease in the bearing property, fluid retention and roughness of the biofilm (Table 1). The AFM topographs suggest full grown biofilm in control compared to treated conditions (Figure 6). Alterations in the physical property under treated conditions led to loosely packed polymers which are not supportive for bacterial adherence; as a result, delicate biofilms are formed. Similarly, a discreet biofilm was visualized under a scanning electron microscopy (Figure 5). The steady decrease of biofilm formation was associated with an increase of extract concentration and about 60% biofilm inhibition was observed with 0.1 mg/ml SJ01 extract. The motility of bacteria plays a vital role in biofilm formation, for which bacteria need to attach to the surface or substratum. They utilize their flagellum driven motility to reach substratum; once attached to the surface, they were spared all around via swimming and swarming, which led to the biofilm formation (O'May and Tufenkji, 2011). The extract of SJ01 inhibits the motility of the *P. aeruginosa* (Figure 7) and thus decreases the possibility of biofilm formation.

A compound 1,2-benzenedicarboxylic acid, diisooctyl ester was identified in the active fraction of the SJ01 extract by GC-MS and ESI (Figure 9). A similar compound, 1,2-benzenedicarboxylic acid bis (2α-methylheptyl) ester, was isolated from *Alcaligenes faecalis* YMF 3.175 and reported to have antibacterial activity against *Escherichia coli* and *Staphylococcus aureus* (Zhu et al., 2011). The antibacterial activity was also reported for 1,2-benzenedicarboxylic acid, mono (2-ethylhexyl) ester isolated from the endophytic fungus *Muscodor tigerii* (Saxena et al., 2015). However, in this study, antibacterial activity was not detected for 1,2-benzenedicarboxylic acid, diisooctyl ester (Figure 4 and Figure S4). Secondary infections caused by *P. aeruginosa* are difficult to eradicate due to their high levels of resistance to most conventional antibiotics. The challenge of combatting the infection becomes more complex due to the ability of the pathogen to form a biofilm matrix which protects bacterial cells from environmental stress as well as antibiotics (Driscoll et al., 2007; Lee and Zhang, 2015). It is the first report of anti-quorum sensing and anti-biofilm activity of 1,2-benzenedicarboxylic acid, diisooctyl ester on *P. aeruginosa* however, a detailed study is required to develop this compound as an anti-pathogenic drug for the treatment of the biofilm forming pathogenic bacteria.

Early colonization on host tissues is initiated by elastase and protease, whereas pyocyanin interferes with multiple cellular functions, chelates iron uptake, and promotes virulence expression (Lau et al., 2004; Stehling et al., 2008). The rhamnolipids facilitate surface motility of *P. aeruginosa* for biofilm formation and are also involved in the dispersal of mature biofilm (O'May and Tufenkji, 2011). Thus, the pathogenicity of *P. aeruginosa* depends on the virulence factor, and pyocyanin plays a key role in this infection (Lau et al., 2004). It was observed that pyocyanin production decreased by about 70 and 55% in strain PAO1 and PAH, respectively, by SJ01 extract (Figure 8). Furthermore, rhamnolipid, protease, and elastase are also regarded as important indicators for quorum sensing (Sarabhai et al., 2013). About 85 and 67% reduction of rhamnolipid production was noticed for *P. aeruginosa* PAO1 and PAH, respectively; however, a significant decrease (24–35%) was observed for protease and elastase activity by SJ01 extract (Figure 8). The production and activity of virulence factors is controlled by the *las* and *rhl* regulatory system in *P. aeruginosa* (De Kievit and Iglewski, 2000; Kohler et al., 2000).

The GeneChip probe array is a powerful tool for monitoring transcriptional regulation of any organism. The array used in this study represents the annotated genome of *P. aeruginosa* strain PAO1 and includes 5,549 protein-coding sequences, 18 tRNA genes, a representative of the ribosomal RNA cluster and 117 genes present in strains other than PAO1. The microarray analysis showed the differential expression of 1,434 genes and revealed that a large number of genes are directly or indirectly involved in biofilm formation (Figure 10, Table 2, and Table S1). Most of these genes are involved in quorum sensing, virulence, motility, and transport. Transcriptional regulators and hypothetical proteins were also differentially expressed and thus may play an important role in biofilm formation. The key genes, *LasI, LasR, RhlI*, and *RhlR*, were down-regulated in *P. aeruginosa* compared to the control (Figure 10).

The *las* regulatory system of *P. aeruginosa* consists of the LasI synthase protein and LasR transcriptional regulator. LasI is essential for the production of the AHL signal molecule N-(3-oxododecanoyl)-l-homoserine lactone (3O-C_12_-HSL), and LasR requires 3O-C_12_-HSL to become an active transcription factor (Gambello and Iglewski, 1991; Pearson et al., 1994; Kiratisin et al., 2002). A second QS system (of *P. aeruginosa*), *rhl*, is also comprised of the RhlI and RhlR proteins. RhlI synthase produces the AHL *N*-butyryl-l-homoserine lactone (C_4_-HSL) and the transcriptional regulator RhlR becomes activated when complexed with C_4_-HSL (Ochsner et al., 1994; Pearson et al., 1995). Both *lasR* and *rhlR* regulate the expression of several genes and activity including, pyocyanin, rhamnolipid, elastase, protease, and motility.

**Figure 11:**
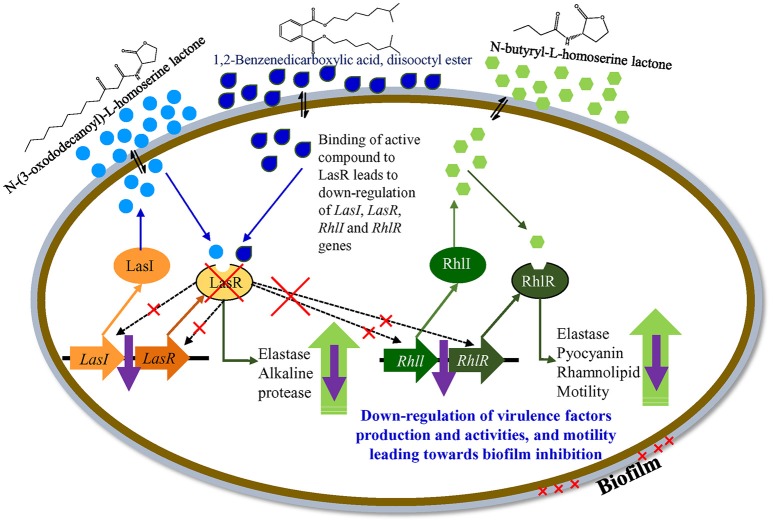
A hypothetical model explaining transcriptional regulation of QSI in *P. aeruginosa*.

Based on the differential gene expression (microarray and qRT-PCR) of quorum sensing key regulatory gene(s) a theoretical model for the transcriptional regulatory mechanism in *P. aeruginosa* was inferred (Figure 11). The proposed model is just a schematic representation (based on available literature) in the form of a hypothetical model explaining transcriptional regulation of QSI in *P. aeruginosa*. However, a detailed study is needed to confirm the exact role of the identified compound in the QSI regulation mechanism. It was hypothesized that the identified compound 1,2-benzenedicarboxylic acid, diisooctyl ester (showing structural similarity with AHL) may compete with AHL and bind to LasR. Binding with LasR down-regulates the protease and elastase activity, along with expression of the *rhl* regulatory system. Down-regulation of the *rhl* QS system leads to the lower activity of pyocyanin and rhamnolipid production along with elastase, protease, and motility. Results indicate that the active compound may decrease the production of virulence factors through transcriptional regulation of the expression of *las* and *rhl* QS systems.

## Conclusion

A bacterium, *D. tsuruhatensis* SJ01, isolated from the rhizosphere of *C. laevigatus* showed anti-quorum sensing and anti-biofilm activities. Furthermore, SJ01 extract does not possess anti-bacterial properties. A compound 1,2-benzenedicarboxylic acid, diisooctyl ester was identified as a probable active compound in the bacterial fraction. The compound inhibits the biofilm formation of clinical isolate *P. aeruginosa* PAO1 and human pathogenic strain *P. aeruginosa* PAH by decreasing the swimming and swarming motility and regulating virulence factors such as pyocyanin, rhamnolipid, elastase, and protease. The compound may intervene in the QS system of *P. aeruginosa* and down-regulate the gene(s) responsible for the quorum sensing mechanism. Our results demonstrate that the active compound may target the QS systems. Targeting a QS system is important for therapeutics, and this may be used for the effective treatment of biofilm-related infection. The inhibitor may be a potent drug for the eradication of *P. aeruginosa* infections, and the active compound has the potential to be developed as an anti-pathogenic drug; however, a detailed study is still needed to investigate potential pharmaceutical applications.

## Author contributions

Conceived and designed the experiments: AM and BJ; Performed the experiments: VS; Analyzed the data: VS and AM; Wrote the manuscript: AM and VS.

### Conflict of interest statement

The authors declare that the research was conducted in the absence of any commercial or financial relationships that could be construed as a potential conflict of interest.

## Supplementary material

Figure S1 Antibacterial disc diffusion assay of *D. tsuruhatensis* SJ01 against *C. violaceum* CV026. The Mueller-Hinton agar (MHA) plate containing reference strain *C. violaceum* CV026 were tested for antibacterial activity of *D. tsuruhatensis* SJ01. Strain SJ01 represents the culture (5 μl) and the antibiotic tobramycin (5 μl) was used as a positive control.

Figure S2 Phylogenetic position of *D. tsuruhatensis* SJ01 (KX130769) with taxonomic neighbors. Numbers at nodes are percentage bootstrap values. The phylogenetic tress was computed using the maximum composite likelihood method and are in the units of the number of base substitutions per site. All positions containing gaps and missing data were eliminated by the complete deletion option. Phylogenetic analysis was conducted in MEGA (ver 6). Bar indicates 0.005 substitutions per nucleotide position.

Figure S3 Whole cell fatty acid profiling of the bacterium *D. tsuruhatensis* SJ01. The whole cell fatty acid profile of strain SJ01 was performed by GC coupled with MIDI. The name of the fatty acids was assigned on the basis of corresponding fatty acids of RTSBA6 6.10 library match.

Figure S4 Effect of extract of *D. tsuruhatensis* SJ01 extract on planktonic cell growth of *P. aeruginosa*. Different concentration of bacterial extracts (SJ01; 0.01−0.1 mg/ml) was tested against biofilm forming reference strain *P. aeruginosa* PAO1 and pathogenic strain *P. aeruginosa* PAH. Tests without extract and with methanol were considered as control and negative control, respectively.

Figure S5 Effect of extract (0.1 mg/ml) of *D. tsuruhatensis* SJ01 extract on the growth curve of *P. aeruginosa*. Bacterial extracts (SJ01; 0.1 mg/ml) was tested for effect on growth of biofilm forming reference strain *P. aeruginosa* PAO1 and pathogenic strain *P. aeruginosa* PAH. The OD was taken up to 24 h at 600 nm using spectrophotometer. Growth of bacteria without treatment of extract (SJ01) was considered control.

Figure S6 Antibacterial disc diffusion assay of *D. tsuruhatensis* SJ01 against *P. aeruginosa*. Clinical isolates of *P. aeruginosa* PAO1 and PAH were tested for antibacterial activity of *D. tsuruhatensis* SJ01 extract. The bacterial extract did not show any antibacterial activity against the clinical isolates *P. aeruginosa*.

Figure S7 A microarray scattered plot showing differential expression of genes. Up- and down- regulation of genes are indicated by blue and green colored marks, respectively. The analysis was performed in expression console and transcriptome analysis console, and genes exhibiting significant fold expression (ANOVA p < 0.05) were considered for the study.

Table S1 Total transcripts that differentially expressed (up- or down- regulated) in *P. aeruginosa* PAO1, treated with bacterial (*D. tsuruhatensis* SJ01) active fraction (C18-100; containing 1,2-benzenedicarboxylic acid, diisooctyl ester) compared with control (untreated PAO1 strain). Array-Express accession E-MTAB-5693.
