# Corrigendum: Anti-quorum Sensing and Anti-biofilm Activity of *Delftia tsuruhatensis* Extract by Attenuating the Quorum Sensing-Controlled Virulence Factor Production in *Pseudomonas aeruginosa*

**DOI:** 10.3389/fcimb.2019.00308

**Published:** 2019-08-23

**Authors:** Vijay K. Singh, Avinash Mishra, Bhavanath Jha

**Affiliations:** Marine Biotechnology and Ecology Division, CSIR-Central Salt and Marine Chemicals Research Institute, Bhavnagar, India

**Keywords:** anti-biofilm, anti-quorum, microarray, quorum network, quorum quenching, quorum sensing, virulence factors

In the original article, there was an error. “**SJ16 (1.0 mg/ml)”** should have read as **“SJ01 (0.1 mg/ml)**.**”**

A correction has been made to ***Materials and Methods, Virulence Factor Analysis*,** ***Paragraph 1***:

The effect of bacterial extracts (SJ01; 0.1 mg/ml) was studied on the production of virulence factors of reference *P. aeruginosa* strains by quantifying pyocyanin and rhamnolipid, and analyzing elastase and protease activities. Briefly, *P. aeruginosa* PAO1 and *P. aeruginosa* PAH were grown overnight in 5 ml of PB medium (20 g/l peptone, 1.4 g/l MgCl_2_ and 10 g/l K_2_SO_4_) supplemented with extract of strain SJ01 (0.1 mg/ml) and without extract (control) at 37°C (180 rpm). The culture was centrifuged at 10,000 × g for 10 min, and pyocyanin was extracted first from the supernatant in 3 ml of chloroform, followed by 1 ml of 0.2 N HCl. The absorbance was measured spectrophotometrically at 520 nm (Essar et al., 1990).

In the original article, there was an error. **“After incubation, the mixture was centrifuged at 30,000** **×** **g for 10 min”** should have read as follows: **“After incubation, the mixture was centrifuged at 3,000** **× g for 10 min**.**”**

A correction has been made to ***Materials and Methods, Virulence Factor Analysis*,** ***Paragraph 2***:

For rhamnolipid, reference strains (*P. aeruginosa*) were grown in nutrient broth supplemented with bacterial extract (SJ01; 0.1 mg/ml) or without extract (control). The culture was centrifuged at 10,000 × g for 10 min, supernatants were collected, acidified with HCl (to pH 2) and absorbance was measured at 570 nm (McClure and Schiller, 1992). Supernatants (750 μl) of overnight grown (with 0.1 mg/ml or without extract of strain SJ01) *P. aeruginosa* were incubated with 250 μl elastin Congo-red solution (5 mg/ml in 0.1 M tris-HCl pH 8; 1 mM CaCl_2_) at 37°C, 180 rpm for 16 h. After incubation, the mixture was centrifuged at 3,000 × g for 10 min, and absorbance was measured at 490 nm for elastase activity (Zhu et al., 2002). For protease activity, supernatant (400 μl) was incubated with an equal volume of 2% azocasein solution (prepared in 50 mM phosphate buffer saline, pH 7) at 37°C for 1 h. The reaction was stopped by adding 500 μl of 10% trichloroacetic acid (TCA), and reaction mix was centrifuged at 8,000 g for 5 min to remove residual azocasein. The absorbance of the supernatant was read at 400 nm (Adonizio et al., 2008).

The authors apologize for this error and state that this does not change the scientific conclusions of the article in any way. The original article has been updated.
