# Supplementary material for: Anti-quorum Sensing and Anti-biofilm Activity of Delftia tsuruhatensis Extract by Attenuating the Quorum Sensing-Controlled Virulence Factor Production in Pseudomonas aeruginosa
Source: Front Cell Infect Microbiol. 2017 Jul 26;7:337. doi: 10.3389/fcimb.2017.00337 (PMC5526841; doi:10.3389/fcimb.2017.00337)
Supplement: Figure S1 — Antibacterial disc diffusion assay of D. tsuruhatensis SJ01 against C. violaceum CV026. The Mueller-Hinton agar (MHA) plate containing reference strain C. violaceum CV026 were tested for antibacterial activity of D. tsuruhatensis SJ01. Strain SJ01 represents the culture (5 μl) and the antibiotic tobramycin (5 μl) was used as a positive control. [file Image1.PDF]

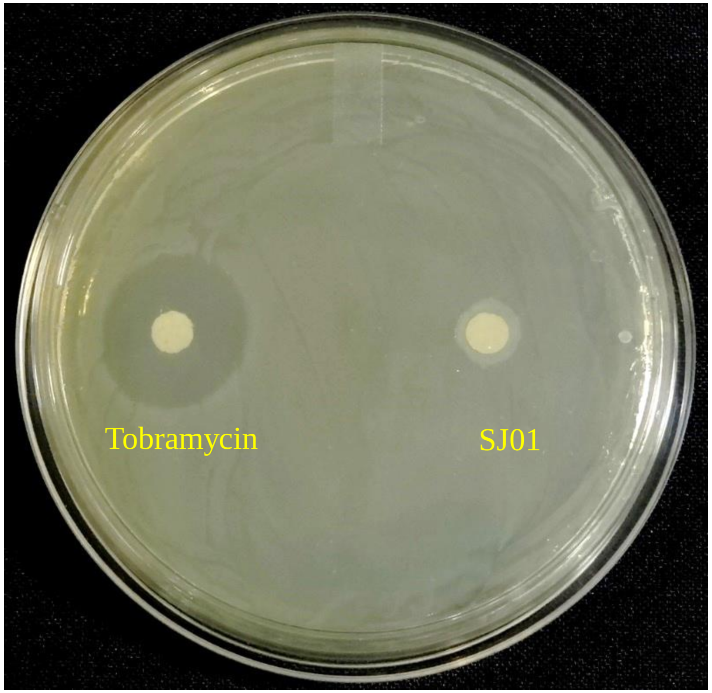

Figure S1: Antibacterial disc diffusion assay of *D. tsuruhatensis* SJ01 against *C. violaceum* CV026. The Mueller-Hinton agar (MHA) plate containing reference strain *C. violaceum* CV026 were tested for antibacterial activity of *D. tsuruhatensis* SJ01. Strain SJ01 represents the culture (5 µl) and the antibiotic tobramycin (5 µl) was used as a positive control.
