# Supplementary material for: Anti-quorum Sensing and Anti-biofilm Activity of Delftia tsuruhatensis Extract by Attenuating the Quorum Sensing-Controlled Virulence Factor Production in Pseudomonas aeruginosa
Source: Front Cell Infect Microbiol. 2017 Jul 26;7:337. doi: 10.3389/fcimb.2017.00337 (PMC5526841; doi:10.3389/fcimb.2017.00337)
Supplement: Figure S2 — Phylogenetic position of D. tsuruhatensis SJ01 (KX130769) with taxonomic neighbors. Numbers at nodes are percentage bootstrap values. The phylogenetic tress was computed using the maximum composite likelihood method and are in the units of the number of base substitutions per site. All positions containing gaps and missing data were eliminated by the complete deletion option. Phylogenetic analysis was conducted in MEGA (ver 6). Bar indicates 0.005 substitutions per nucleotide position. [file Image2.PDF]

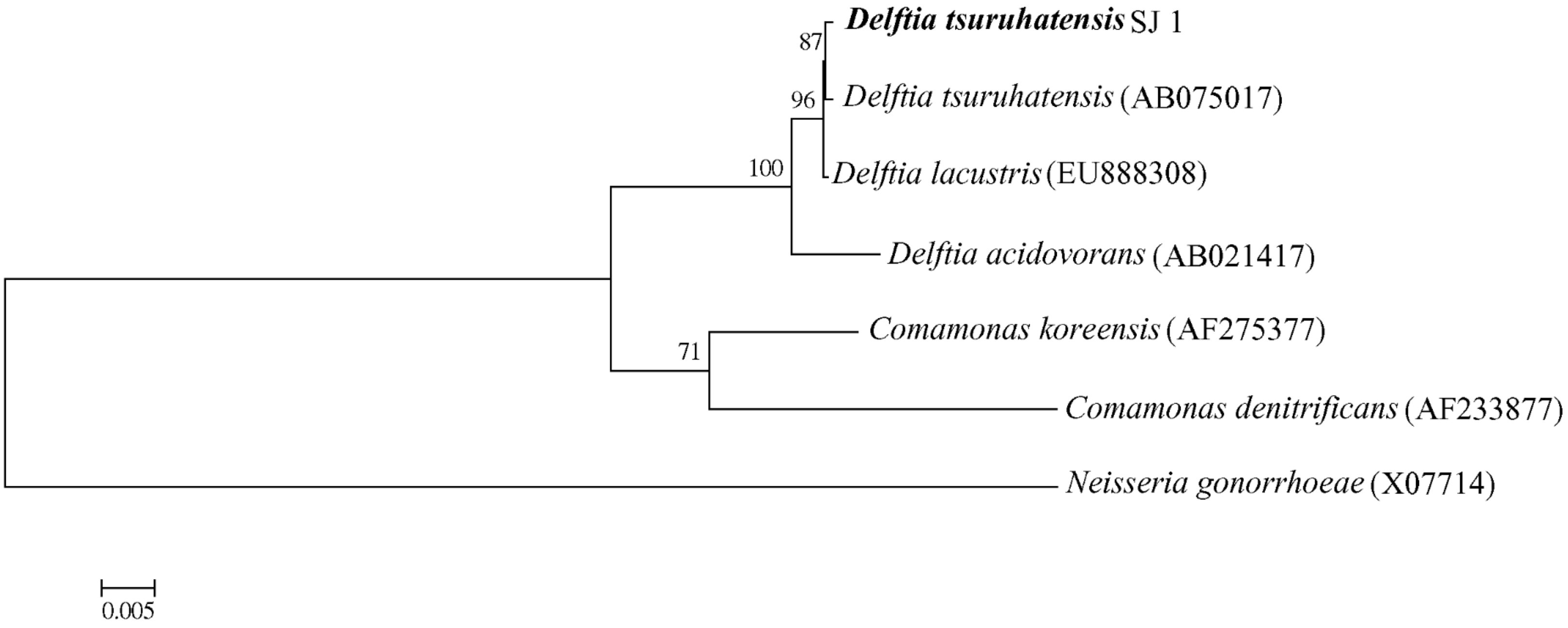

Figure S2: Phylogenetic position of *D. tsuruhatensis* SJ01 (KX130769) with taxonomic neighbors. Numbers at nodes are percentage bootstrap values. The phylogenetic tress was computed using the maximum composite likelihood method and are in the units of the number of base substitutions per site. All positions containing gaps and missing data were eliminated by the complete deletion option. Phylogenetic analysis was conducted in MEGA (ver 6). Bar indicates 0.005 substitutions per nucleotide position.
