# Supplementary material for: Anti-quorum Sensing and Anti-biofilm Activity of Delftia tsuruhatensis Extract by Attenuating the Quorum Sensing-Controlled Virulence Factor Production in Pseudomonas aeruginosa
Source: Front Cell Infect Microbiol. 2017 Jul 26;7:337. doi: 10.3389/fcimb.2017.00337 (PMC5526841; doi:10.3389/fcimb.2017.00337)
Supplement: Figure S3 — Whole cell fatty acid profiling of the bacterium D. tsuruhatensis SJ01. The whole cell fatty acid profile of strain SJ01 was performed by GC coupled with MIDI. The name of the fatty acids was assigned on the basis of corresponding fatty acids of RTSBA6 6.10 library match. [file Image3.PDF]

Central Salt and Marine Chemicals Research Institute

Volume: DATA1 File: E151155.03A Samp Ctr: 16 ID Number: 895
Type: Samp Bottle: 14 Method: RTSBA6
Sample ID: SJ01 Sherlock Version 6.1 [S/N 160321]

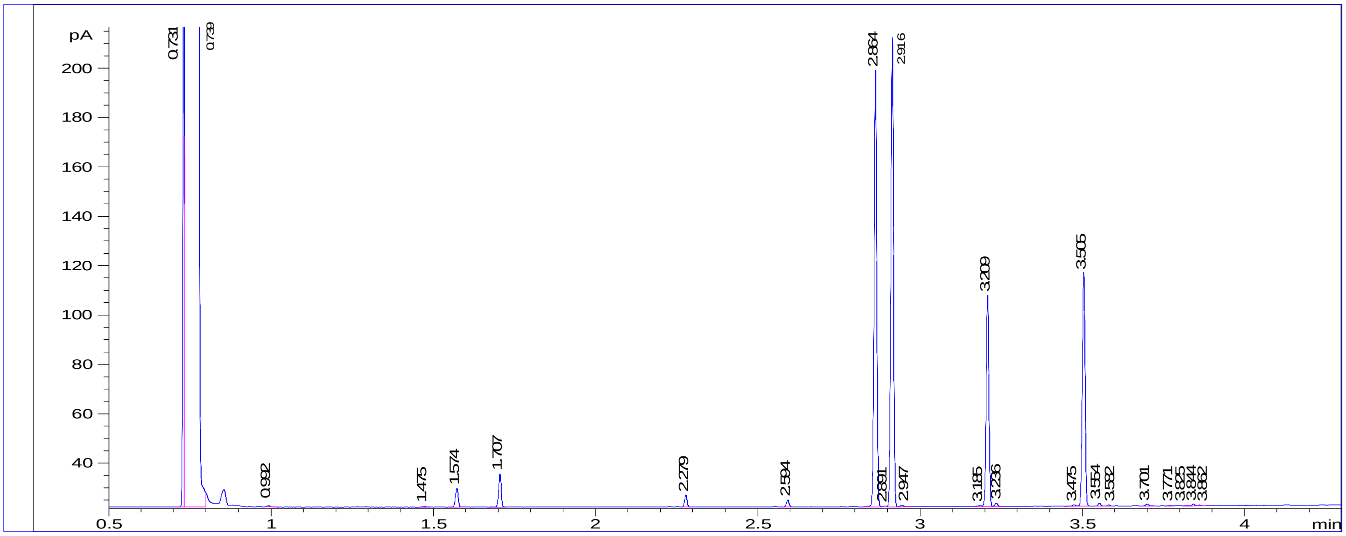

RT	Response	Ar/Ht	RFact	ECL	Peak Name	Percent	Comment1	Comment2
0.7305	150641	0.005	----	6.5803		----	< min rt	
0.7393	9.897E+8	0.019	----	6.6359	SOLVENT PEAK	----	< min rt	
0.9921	502	0.007	----	8.2465		----	< min rt	
1.4747	559	0.010	----	11.0386		----		
1.5739	8933	0.009	1.050	11.4508	10:0 3OH	1.48	ECL deviates 0.003	
1.7067	14713	0.008	1.031	12.0015	12:0	2.39	ECL deviates 0.002	Reference 0.013
2.2794	5147	0.008	0.981	13.9963	14:0	0.79	ECL deviates -0.004	Reference 0.013
2.5938	3190	0.009	----	14.9981	15:0	----	ECL deviates -0.002	
2.8640	200683	0.009	0.955	15.8402	Sum In Feature 3	30.15	ECL deviates 0.000	16:1 w7c/16:1 w6c
2.8915	329	0.008	0.954	15.9258	16:1 w5c	0.05	ECL deviates -0.002	
2.9158	217443	0.009	0.953	16.0017	16:0	32.62	ECL deviates 0.002	Reference 0.018
2.9469	966	0.010	----	16.0988		----		
3.1852	734	0.012	0.947	16.8419	17:1 w7c	0.11	ECL deviates 0.006	
3.2093	100507	0.009	0.947	16.9174	17:0 cyclo	14.97	ECL deviates 0.002	
3.2358	1554	0.009	0.946	17.0001	17:0	0.23	ECL deviates 0.000	Reference 0.014
3.4754	829	0.010	0.943	17.7550	Sum In Feature 5	0.12	ECL deviates -0.001	18:2 w6,9c/18:0 ante
3.5055	111877	0.010	0.943	17.8497	Sum In Feature 8	16.60	ECL deviates 0.002	18:1 w7c
3.5539	1533	0.010	0.942	18.0027	18:0	0.23	ECL deviates 0.003	Reference 0.012
3.5821	745	0.010	----	18.0939		----		
3.7008	1191	0.011	----	18.4775		----		
3.7713	406	0.010	----	18.7056		----		
3.8253	393	0.009	0.941	18.8802	Sum In Feature 7	0.06	ECL deviates -0.007	19:0 cyclo w10c/19w6
3.8438	913	0.009	0.941	18.9401	19:0 cyclo w8c	0.14	ECL deviates 0.008	
3.8616	502	0.010	0.941	18.9978	19:0	0.07	ECL deviates -0.002	Reference 0.003
----	200683	---	----	----	Summed Feature 3	30.15	16:1 w7c/16:1 w6c	16:1 w6c/16:1 w7c
----	829	---	----	----	Summed Feature 5	0.12	18:0 ante/18:2 w6,9c	18:2 w6,9c/18:0 ante
----	393	---	----	----	Summed Feature 7	0.06	19:1w7c/19:1 w6c	19:1 w6c/w7c/19cy
----	-----	---	----	----		----	19:0 cyclo w10c/19w6	
----	111877	---	----	----	Summed Feature 8	16.60	18:1 w7c	18:1 w6c

ECL Deviation: 0.004 Reference ECL Shift: 0.013 Percent Named: 99.42%
Total Response: 669958 Total Named: 666090 Total Amount: 635487

Figure S3: Whole cell fatty acid profiling of the bacterium *D. tsuruhatensis* SJ01. The whole cell fatty acid profile of strain SJ01 was performed by GC coupled with MIDI. The name of the fatty acids was assigned on the basis of corresponding fatty acids of RTSBA6 6.10 library match.
