# Supplementary material for: Anti-quorum Sensing and Anti-biofilm Activity of Delftia tsuruhatensis Extract by Attenuating the Quorum Sensing-Controlled Virulence Factor Production in Pseudomonas aeruginosa
Source: Front Cell Infect Microbiol. 2017 Jul 26;7:337. doi: 10.3389/fcimb.2017.00337 (PMC5526841; doi:10.3389/fcimb.2017.00337)
Supplement: Figure S4 — Effect of extract of D. tsuruhatensis SJ01 extract on planktonic cell growth of P. aeruginosa. Different concentration of bacterial extracts (SJ01; 0.01−0.1 mg/ml) was tested against biofilm forming reference strain P. aeruginosa PAO1 and pathogenic strain P. aeruginosa PAH. Tests without extract and with methanol were considered as control and negative control, respectively. [file Image4.PDF]

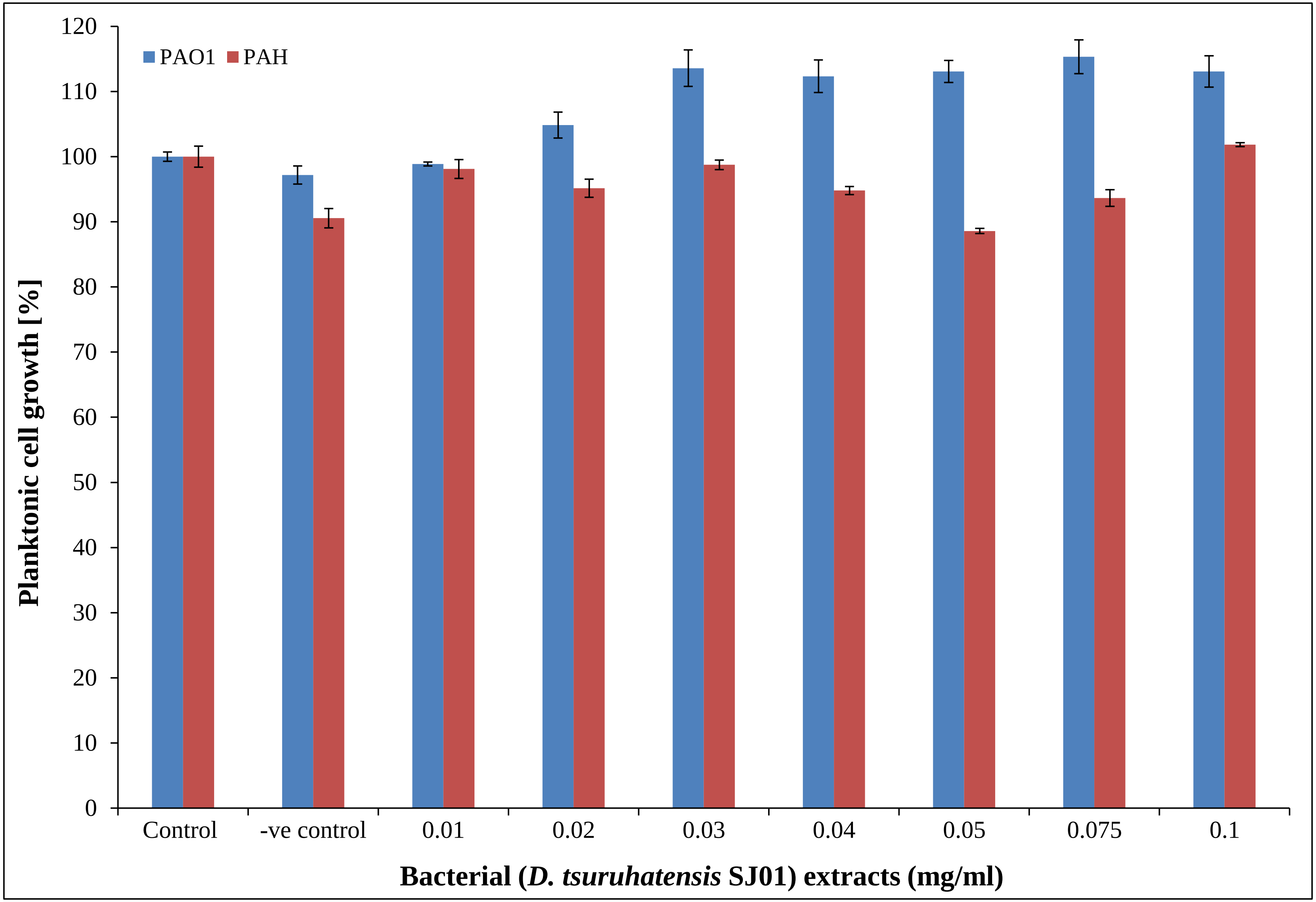

Figure S4: Effect of extract of *D. tsuruhatensis* SJ01 extract on planktonic cell growth of *P. aeruginosa*. Different concentration of bacterial extracts (SJ01; 0.01-0.1 mg/ml) was tested against biofilm forming reference strain *P. aeruginosa* PAO1 and pathogenic strain *P. aeruginosa* PAH. Tests without extract and with methanol were considered as control and negative control, respectively.
