# Supplementary material for: Anti-quorum Sensing and Anti-biofilm Activity of Delftia tsuruhatensis Extract by Attenuating the Quorum Sensing-Controlled Virulence Factor Production in Pseudomonas aeruginosa
Source: Front Cell Infect Microbiol. 2017 Jul 26;7:337. doi: 10.3389/fcimb.2017.00337 (PMC5526841; doi:10.3389/fcimb.2017.00337)
Supplement: Figure S5 — Effect of extract (0.1 mg/ml) of D. tsuruhatensis SJ01 extract on the growth curve of P. aeruginosa. Bacterial extracts (SJ01; 0.1 mg/ml) was tested for effect on growth of biofilm forming reference strain P. aeruginosa PAO1 and pathogenic strain P. aeruginosa PAH. The OD was taken up to 24 h at 600 nm using spectrophotometer. Growth of bacteria without treatment of extract (SJ01) was considered control. [file Image5.PDF]

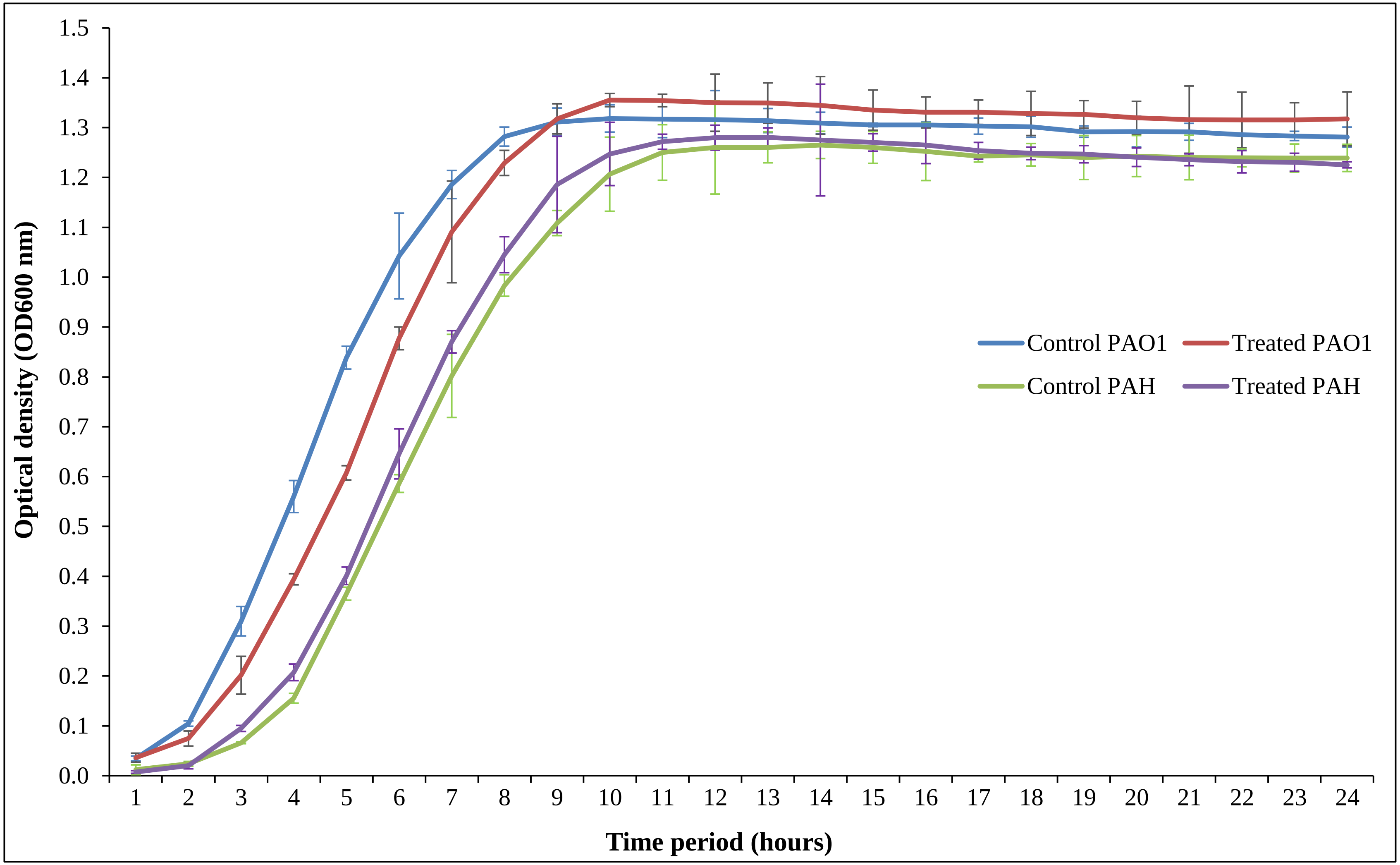

Figure S5: Effect of extract (0.1 mg/ml) of *D. tsuruhatensis* SJ01 extract on the growth curve of *P. aeruginosa*. Bacterial extracts (SJ01; 0.1 mg/ml) was tested for effect on growth of biofilm forming reference strain *P. aeruginosa* PAO1 and pathogenic strain *P. aeruginosa* PAH. The OD was taken up to 24 h at 600 nm using spectrophotometer. Growth of bacteria without treatment of extract (SJ01) was considered control.
