# Supplementary material for: Anti-quorum Sensing and Anti-biofilm Activity of Delftia tsuruhatensis Extract by Attenuating the Quorum Sensing-Controlled Virulence Factor Production in Pseudomonas aeruginosa
Source: Front Cell Infect Microbiol. 2017 Jul 26;7:337. doi: 10.3389/fcimb.2017.00337 (PMC5526841; doi:10.3389/fcimb.2017.00337)
Supplement: Figure S6 — Antibacterial disc diffusion assay of D. tsuruhatensis SJ01 against P. aeruginosa. Clinical isolates of P. aeruginosa PAO1 and PAH were tested for antibacterial activity of D. tsuruhatensis SJ01 extract. The bacterial extract did not show any antibacterial activity against the clinical isolates P. aeruginosa. [file Image6.PDF]

***P. aeruginosa* PAO1** ***P. aeruginosa* PAH**

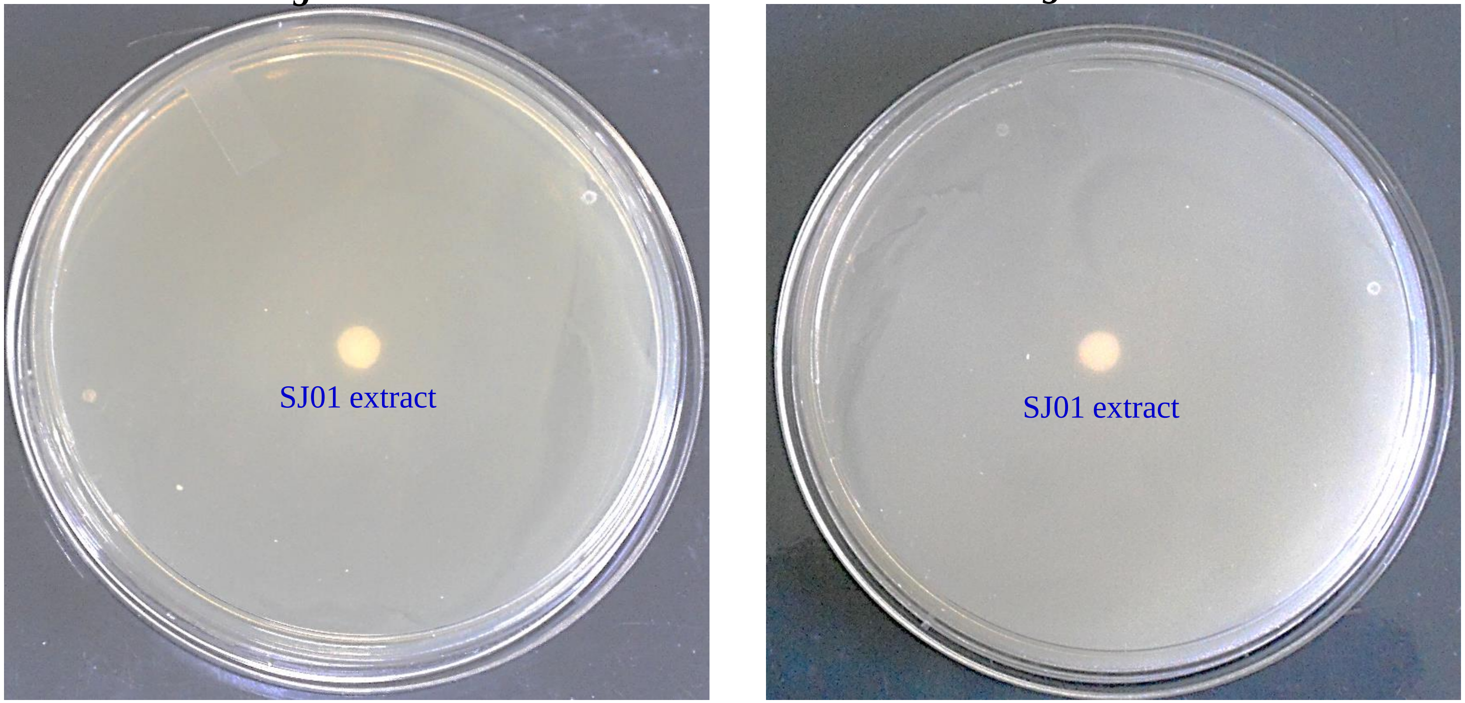

Figure S6: Antibacterial disc diffusion assay of *D. tsuruhatensis* SJ01 against *P. aeruginosa*. Clinical isolates of *P. aeruginosa* PAO1 and PAH were tested for antibacterial activity of *D. tsuruhatensis* SJ01 extract. The bacterial extract did not show any antibacterial activity against the clinical isolates *P. aeruginosa*.
