# Supplementary material for: Anti-quorum Sensing and Anti-biofilm Activity of Delftia tsuruhatensis Extract by Attenuating the Quorum Sensing-Controlled Virulence Factor Production in Pseudomonas aeruginosa
Source: Front Cell Infect Microbiol. 2017 Jul 26;7:337. doi: 10.3389/fcimb.2017.00337 (PMC5526841; doi:10.3389/fcimb.2017.00337)
Supplement: Figure S7 — A microarray scattered plot showing differential expression of genes. Up- and down- regulation of genes are indicated by blue and green colored marks, respectively. The analysis was performed in expression console and transcriptome analysis console, and genes exhibiting significant fold expression (ANOVA p < 0.05) were considered for the study. [file Image7.PDF]

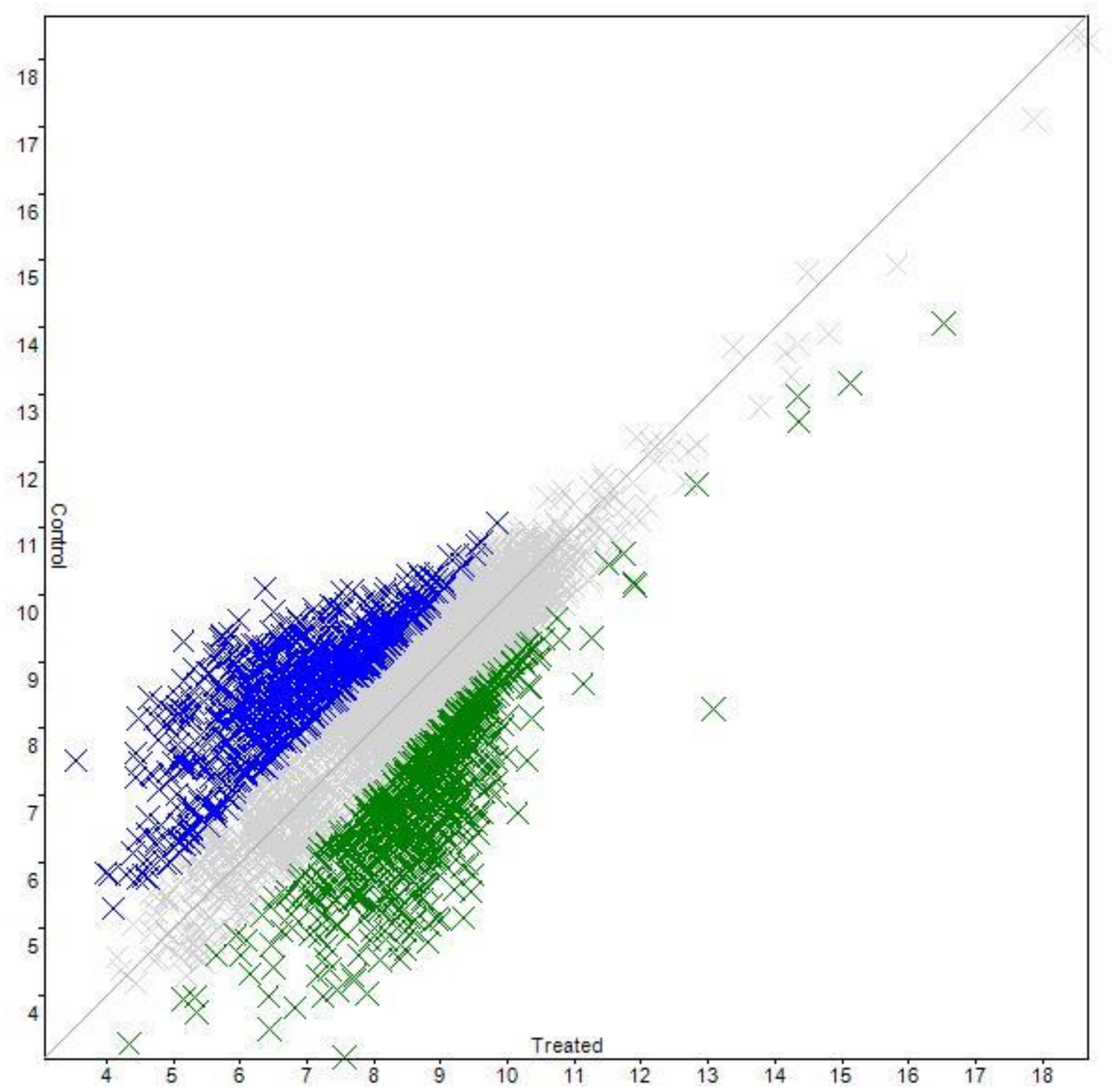

Figure S7: A microarray scattered plot showing differential expression of genes. Up- and down- regulation of genes are indicated by blue and green colored marks, respectively. The analysis was performed in expression console and transcriptome analysis console, and genes exhibiting significant fold expression (ANOVA p-value <0.05) were considered for the study.
